# Electronic Patient Reporting of Adverse Events and Quality of Life: A Prospective Feasibility Study in General Oncology

**DOI:** 10.1200/OP.20.00118

**Published:** 2020-08-27

**Authors:** Fiona Kennedy, Kate Absolom, Beverly Clayton, Zoe Rogers, Kathryn Gordon, Elaine O’Connell Francischetto, Jane M. Blazeby, Julia Brown, Galina Velikova

**Affiliations:** ^1^ Section of Patient-Centered Outcomes Research, Patient Reported Outcomes Group, Leeds Institute of Medical Research at St James’s, University of Leeds, Leeds, United Kingdom; ^2^ Clinical Trial Research Unit, Leeds Institute of Clinical Trials Research, University of Leeds, Leeds, United Kingdom; ^3^ Medical Research Council ConDuCT-II Hub and the Bristol Biomedical Research Centre, Population Health Sciences Bristol Medical School, University of Bristol, Bristol, United Kingdom

## Abstract

Adverse event (AE) reporting is essential in clinical trials. Clinician interpretation can result in under-reporting; therefore, the value of patient self-reporting has been recognized. The National Cancer Institute has developed a Patient-Reported Outcomes version of the Common Terminology Criteria for Adverse Events (PRO-CTCAE) for direct patient AE reporting. A nonrandomized prospective cohort feasibility study aimed to explore the compliance and acceptability of an electronic (Internet or telephone) system for collecting patient self-reported AEs and quality of life (QOL).

**METHODS:**

Oncology patients undergoing treatment (chemotherapy, targeted agents, hormone therapy, radiotherapy, and/or surgery) at 2 hospitals were sent automated weekly reminders to complete PRO-CTCAE once a week and QOL (for a maximum of 12 weeks). Patients had to speak/understand English and have access to the Internet or a touch-tone telephone. Primary outcome was compliance (proportion of expected questionnaires), and recruitment rate, attrition, and patient/staff feedback were also explored.

**RESULTS:**

Of 520 patients, 249 consented (47.9%)—mean age was 62 years, 51% were male, and 70% were married—and 230 remained on the study at week 12. PRO-CTCAE was completed at 2,301 (74.9%) of 3,074 timepoints and QOL at 749 (79.1%) of 947 timepoints. Individual weekly/once every 4 weeks compliance reduced over time but was more than 60% throughout. Of 230 patients, 106 (46.1%) completed 13 or more PRO-CTCAE, and 136 (59.1%) of 230 patients completed 4 QOL questionnaires. Most were completed on the Internet (82.3%; mean age, 60.8 years), which was quicker, but older patients preferred the telephone option (mean age, 70.0 years). Positive feedback was received from patients and staff.

**CONCLUSION:**

Self-reporting of AEs and QOL using an electronic home-based system is feasible and acceptable. Implementation of this approach in cancer clinical trials may improve the precision and accuracy of AE reporting.

## INTRODUCTION

As more cancer treatments with innovative modes of action are evaluated via clinical trials, the need to monitor and record adverse events (AEs)^1^ increases to ensure safety and guide prescribing and patient information.^2^ The Common Terminology Criteria for Adverse Events (CTCAE)^3^ is the standard method of reporting AEs, allowing clinicians to uniformly classify AEs and their severity, but its focus is on safety rather than exploring patient experiences.^4^ Clinician interpretation can result in under-reporting of lower-grade morbidity and severity downgrading^2,5,6^; therefore, patient self-reporting is a complementary alternative.^7,8^ The CTCAE system is suitable for acute AEs when patients receive regular oncologist review; however, many new treatments are outpatient-based oral therapies that require less frequent reviews. Although these treatments do not result in the acute severe AEs observed with chemotherapy, they often result in low- to moderate-grade prolonged symptoms that significantly impact patients’ lives and treatment adherence.^1,9^ CTCAE is less appropriate for monitoring AEs during these treatments.^7^

Patient-reported outcomes (PROs) enable systematic, subjective measurement of symptoms and experiences. Health-Related Quality of Life (HRQOL) questionnaires are well-established PRO measures used in phase II and III randomized clinical trials.^10^ Implementing PROs to routinely monitor individual patients’ AEs is relatively uncommon, despite accumulating evidence demonstrating improvement in communication, patient well-being, symptom control, and survival when clinicians receive these data.^11-14^

The National Cancer Institute developed the Patient-Reported Outcome version of the CTCAE item bank (PRO-CTCAE) to obtain direct patient AEs in trials.^15^ Technology can help PRO-CTCAE to become a standardized trial approach,^15,16^ and Web-based PRO efficiency and patient acceptability has been demonstrated.^17,18^ Of the United Kingdom population, 90% are regular Internet users; however, 24% of those age 65 years and older reported no Internet use in the past 3 months.^19^ Therefore, a telephone interactive voice response system (IVRS) widens eligibility.

Oncology clinicians, trialists, the pharmaceutical industry, and regulatory authorities recognize the importance of collecting PRO data in drug development and evaluation.^1,10,20,21^ Concerns still exist about potential patient burden and data collection frequency.^22^ There is a pressing need to develop robust systems to collect PRO-CTCAE alongside clinician-reported CTCAE, as well as explore the feasibility of collecting frequent PRO-CTCAE in addition to HRQOL at prespecified trial timepoints where QOL is an important primary or secondary outcome.

This feasibility study evaluated a self-reported electronic system—Internet/IVRS—to collect PRO-CTCAE and HRQOL in a mixed group of oncology patients and to combine the data with a standard clinical trial database. Primary outcome was compliance and secondary outcomes included recruitment rates, Internet/IVRS uptake, attrition, patient and staff feedback, and system testing.

## METHODS

### Patient Population and Study Design

This proof-of-principle, nonrandomized, prospective, cohort feasibility study was conducted in 2 United Kingdom hospitals between August 19, 2014, and October 7, 2015, with 12-week follow-up. The study design modeled a hypothetical clinical trial scenario in which PRO-CTCAE data are collected for safety (real time, as frequently as needed) and HRQOL at predefined timepoints to assess treatment outcomes.

Our aim when defining the study population was to capture a range of patients across the main cancer sites and treatments. We anticipated that this would increase the generalizability of the findings. Consecutive eligible patients were approached. Therefore, the sample target was 210 participants with early or metastatic cancer receiving either chemotherapy, targeted agents, hormone therapy, radiotherapy, or surgery (42 patients per group, allowing for 30% attrition),^23^ plus 42 patients with Eastern Cooperative Oncology Group (ECOG) performance status score of 2 to 3 receiving any treatment. Eligibility criteria included speaking/understanding English and access to a touch-tone telephone and/or Internet. Patients on clinical trials with regular HRQOL assessments were excluded. Written informed consent was obtained from study participants. Ethical approval was granted from the National Health Service Leeds-East Research Ethics Committee (14.YH.0181).

### Study Outcomes

- Compliance was defined as the proportion of completed questionnaires out of expected completions. This was calculated cumulatively for each questionnaire—PRO-CTCAE and HRQOL—and at each expected timepoint weekly (7-day window)/every 4 weeks (28-day window) across gender, system, study group, and age. Multiple completions per week were discounted from compliance calculations. The number of completions per participant was also calculated, including multiple completions.
- Recruitment rate was defined as the proportion of patients who consented to the study relative to eligible patients approached. Demographics—age, gender, diagnosis, and treatment—were compared between consenting patients and declining patients.
- Internet/IVRS uptake was calculated in 2 ways: the proportion who preferred each system at baseline and the proportion who used each system most often during the study.
- Active withdrawal was the predominant attrition outcome, defined as the proportion of participants who actively requested withdrawal from the study out of participants who consented. Any withdrawal reasons were recorded where provided. In addition, passive withdrawal was defined as the proportion of participants who did not complete any electronic questionnaires postbaseline but did not request withdrawal. Passive withdrawals are included in the compliance calculations, but active withdrawals were excluded after the withdrawal date.
- Patient and staff feedback was captured on a paper end-of-study (EOS) questionnaire that explored experiences of the system(s) and staff views.
- System testing was assessed through the documentation of system issues and phone calls and comments from patients and staff. Call and online session time logs were also recorded.

### Electronic System and Questionnaires

Participant registration via the clinical trials research unit (CTRU) registration system provided the trial number and username and password for accessing the electronic system to complete the 2 self-reported questionnaires.

First, 21 PRO-CTCAE items^15^ covering 11 common symptoms (pain and/or discomfort, nausea, vomiting, diarrhea, constipation, headache, mouth/throat sores, fatigue, skin rash, flu-like symptoms, and muscle ache) were available at any time. Second, the European Organization for the Research and Treatment of Cancer Quality of Life Questionnaire C30 (EORTC-QLQ-C30) 30-item cancer-specific measure exploring HRQOL^24^ was available once every 4 weeks.

Holch et al^25^ provide a detailed overview of the Internet (QTool) system. Internet system users were presented with 1 (PRO-CTCAE) or 2 questionnaires (if EORTC-QLQ-C30 was due). The IVRS that was developed specifically for this study by CTRU technicians was a freephone number. To ease the log-in process on the IVRS, all usernames were a 5-digit numerical code, and passwords were a 4-digit numerical code. The same format was used on the Internet system to enable participants to switch between systems if required. Once logged in, IVRS users heard a welcome message, then heard the EORTC-QLQ-C30 questionnaire (if due, every 4 weeks) and/or the PRO-CTCAE. Each question was read out, followed by the response options (eg, “Question number 13. In the last 7 days, what was the SEVERITY of your fatigue, tiredness, or lack of energy at its worst? Press 1 then hash for none, 2 then hash for mild, 3 then hash for moderate.…”) Patients could interrupt the responses with their selected response and could take a break—hang up and re-ring—after the first questionnaire if needed.

A specific application pulled the data from a separate server that held the Internet/QTool data, which was then stored in a specific PRO database on CTRU servers. A program was created to generate/send automated reports to the local research team detailing completions. The PRO database was combined with the standard trial database (Elsevier's MACRO Data Capture System) that contained all the participant clinical record forms for statistical analysis.

### Procedure

Eligible patients were initially approached by clinical/research staff with the study information sheet. Recruitment was undertaken sequentially across the treatment subgroups and stopped once each prespecified target number was reached.

Demographic details and computer use (frequency and perceived confidence) were self-reported at baseline. Clinical data captured from medical records included diagnosis, treatment, ECOG performance status, and treatment changes (discontinuation/new treatments). Baseline electronic questionnaires were completed in clinic, or a brief demonstration was provided and the full questionnaires completed at home on the preferred system. All participants received step-by-step instructions covering both systems. Participants were reminded (by automated e-mail, text, or both) weekly—every 7 days from the registration date—for 12 weeks to complete the electronic questionnaires. EOS paper questionnaires were sent/given once each participant had completed the 12 weeks.

Any deaths or withdrawal requests were actioned promptly (reminders stopped). All participants were reminded that the system was not intended to replace usual care or methods of reporting any symptoms or concerns to their clinical team. However, researchers endeavored to attach a paper report summarizing any recent AEs to participants’ medical notes at any scheduled outpatient visits.

### Analysis

Data analysis was performed using STATA between August 2016 and February 2017. Descriptive analysis of the study outcomes was conducted. Missing data were not imputed. System issues were coded thematically into generic and patient issues.

## RESULTS

### Study Population

**FIG 1.**
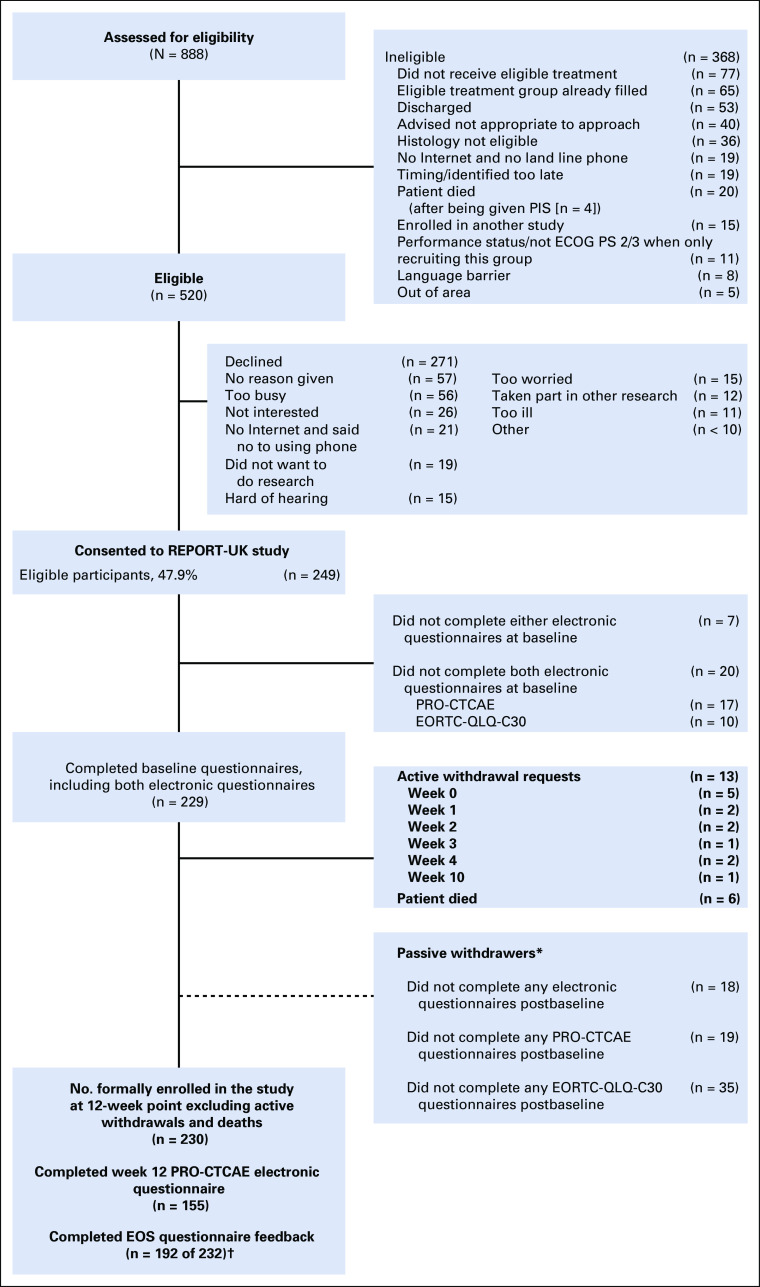
Study recruitment CONSORT diagram. (*) Passive withdrawers are those who did not complete the online questionnaires postbaseline but who did not formally request to be withdrawn. (NOTE. These were included as expected in the compliance calculations.) (†) Two patients who withdrew were sent and completed the (end-of-study (EOS) questionnaire as they had used the systems for some time. ECOG PS, Eastern Cooperative Oncology Group performance status; EORTC-QLQ-C30, European Organization for the Research and Treatment of Cancer Quality of Life Questionnaire C30; PIS, Patient Information Sheet; PRO-CTCAE, Patient Reported Outcomes version of the Common Terminology Criteria for Adverse Events.

Of 888 patients who were assessed for eligibility during the study period, 368 were ineligible, and of 520 approached, 249 consented to the study (recruitment rate 47.9%; Fig 1). Consent rates were higher in targeted agents (57 of 93; 61.3%) and chemotherapy groups (49 of 97; 50.5%), and lower in surgery (42 of 91; 46.2%), radiotherapy (47 of 107; 43.9%), and hormone therapy (54 of 132; 40.9%; χ^2^ = 10.3; *P* = .04).

Participants (mean age, 62.1 years; standard deviation [SD], 13.3 years) were significantly younger than those who declined (mean age, 69.4 years; SD = 12.3 years; t = −6.5; df = 518; *P* < .001). No differences were found by gender (*P* = .46) or disease site (*P* = .08). Of 271 who declined, reasons included being too busy (n = 56; 20.7%), not interested (n = 26; 9.6%), and being too worried/stressed (n = 15; 5.5%). Access to the required technology prevented participation for 21 non-Internet users (who did not want to use IVRS; 7.7%), 15 patients who were hard of hearing (5.5%), and 5 who were unskilled and/or disliked both options (1.8%).

Table 1 lists the demographic and clinical characteristics of the sample. This shows almost similar numbers of male and female patients (n = 128, 51.4%; and n = 121, 48.6%, respectively), and most were married (n = 173; 69.5%) and 124 were retired (49.8%). Breast (n = 57; 22.9%), colorectal (n = 39; 13.7%), and prostate (n = 33; 13.3%) cancer were the most common cancer types. IVRS participants were older (mean age, 70.0 years; SD, 9.9 years; Internet mean age, 60.8 years; SD, 13.7 years), had lower education levels, and more had prostate cancer (12 of 43; 27.9%) compared with Internet users.

**TABLE 1.**
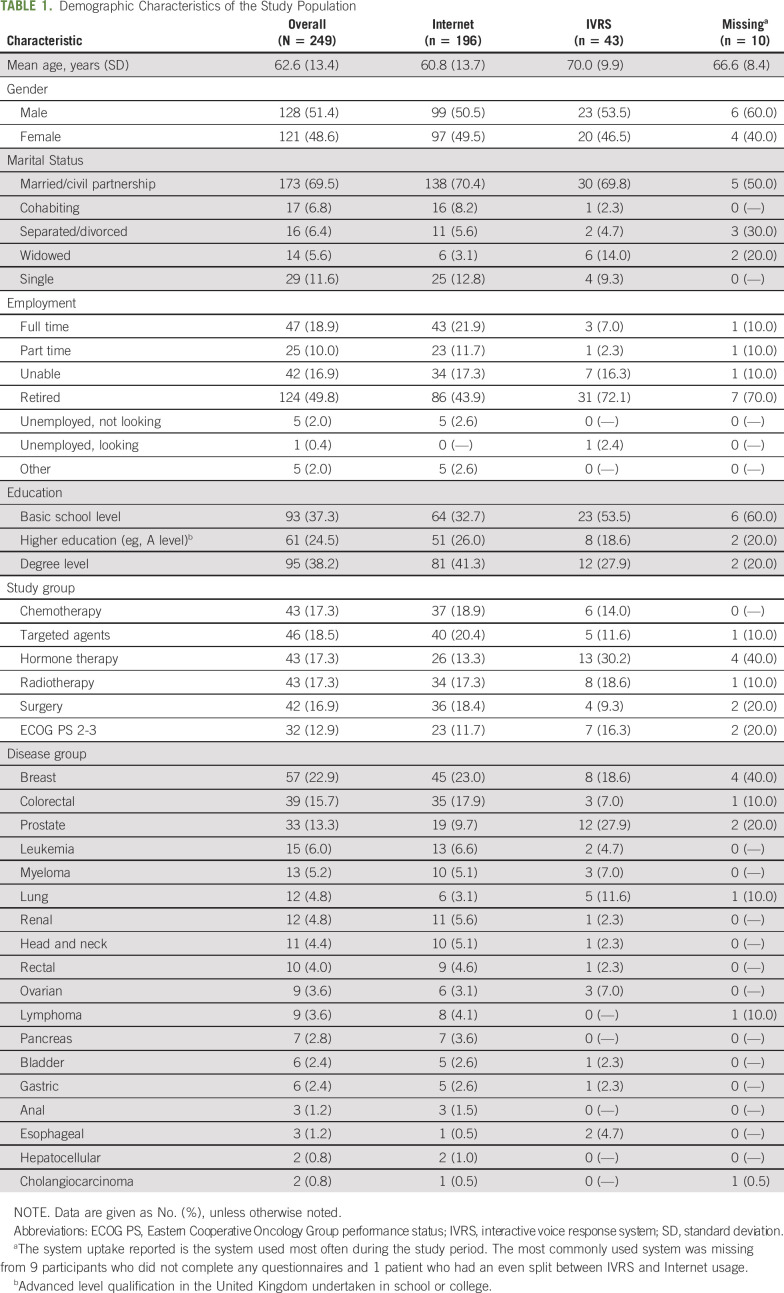
Demographic Characteristics of the Study Population

### Internet/IVRS Uptake

At baseline, 82.3% of participants (205 of 249) indicated they preferred the Internet, whereas 16.9% (42 of 249) preferred the IVRS (1 had no preference and 1 withdrew before registration). Most participants used their preferred system throughout the study period, but a few used both (10 patients when completing the PRO-CTCAE and 6 when completing the EORTC), and 1 patient who preferred IVRS and 3 who preferred the Internet used the opposite system more frequently. Questionnaire completion was quicker on the Internet (PRO-CTCAE median time, 3.0 minutes; interquartile range [IQR], 2.0 to 4.0 minutes; EORTC median time, 6.0 minutes; IQR, 4.0 to 8.0 minutes) compared with IVRS (PRO-CTCAE median time, 7.0 minutes; IQR, 6.1 to 13.6 minutes; EORTC median time, 14.5 minutes; IQR, 13.5 to 15.9 minutes).

### Attrition and Compliance

At week 12, 92.4% of participants (230 of 249) remained on study, with 6 deaths and 13 active withdrawals (5.2%; 5 in week 1, 7 by week 5, and 1 in week 10). Five patients who withdrew were receiving hormone therapy and 8 were across the other treatment groups (ECOG/targeted/radiotherapy, n = 2; chemotherapy/surgery, n =1). Withdrawal reasons included the time required to participate (n = 2), being unwell (n = 2), stopped treatment (n = 1), and computer problems (n = 2). Eighteen participants were classified as passive withdrawals (7.2%), having not completed any electronic questionnaires postbaseline.

**FIG 2.**
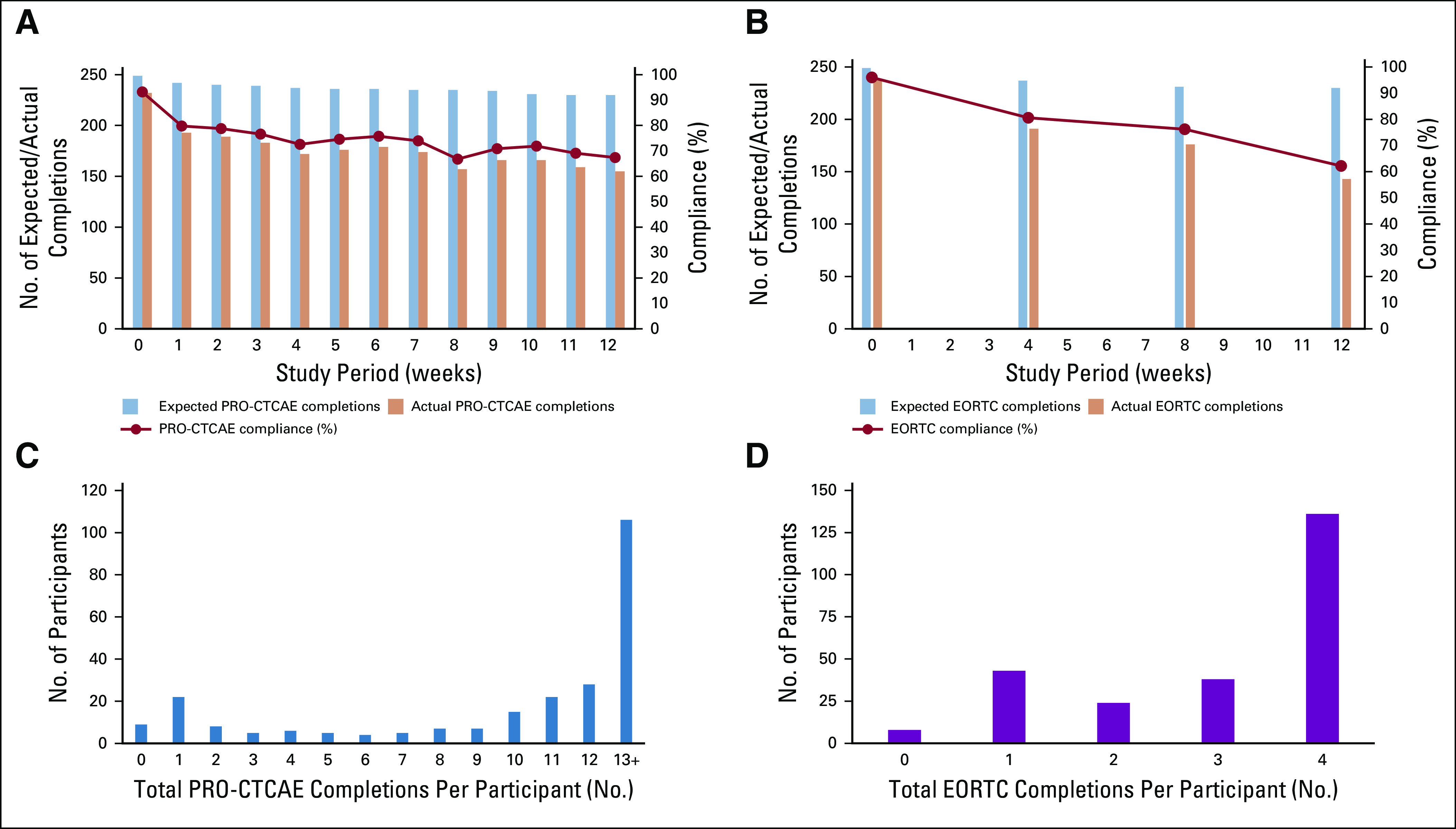
Compliance and number of completions per participant for PRO-CTCAE (Patient Reported Outcomes version of the Common Terminology Criteria for Adverse Events) and EORTC (European Organization for Research and Treatment of Cancer QLQ-C30) questionnaires. (A) PRO-CTCAE weekly compliance across the 12-week study period. (B) EORTC compliance every 4 weeks across the 12-week study period. (C) Total number of PRO-CTCAE completions per participant. (D) Total number of EORTC completions per participant.

Overall combined compliance for both systems was 74.9% for PRO-CTCAE (completed at 2,301 of 3,074 timepoints) and 79.1% for EORTC (completed at 749 of 947 timepoints). Table 2 lists compliance at each timepoint split by gender, system, study group, and age. Individual timepoint compliance (Figs 2A and 2B) shows more than 60% compliance throughout. Completions per participant illustrates that 46.1% (106 of 230) completed 13 or more PRO-CTCAE questionnaires, more than 80% (185 of 230) completed 8 or more, and 59.1% (136 of 230) completed the maximum 4 EORTC questionnaires (Figs 2C and 2D).

**TABLE 2.**
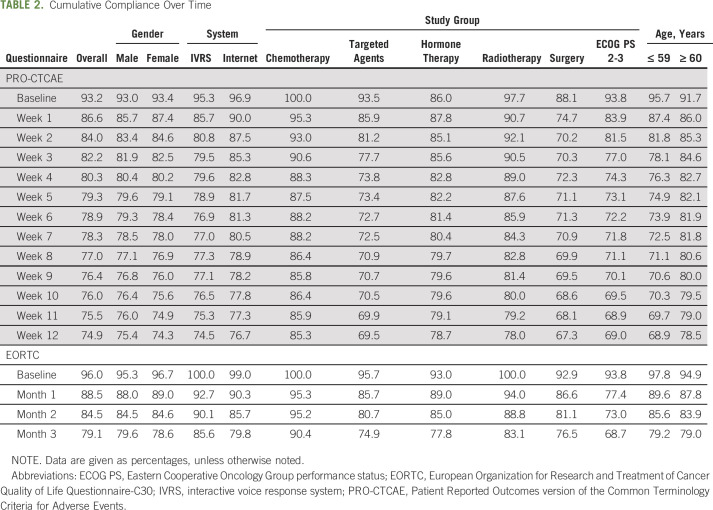
Cumulative Compliance Over Time

Compliance was higher in chemotherapy patients, with week 12 cumulative rates of 85.3% and 90.4% for PRO-CTCAE and EORTC, respectively (Table 2). Surgical participants had the lowest PRO-CTCAE compliance (baseline, 88.1%; week 12 cumulative, 67.3%). ECOG participants had lower EORTC compliance (baseline, 93.8%; month 3 cumulative, 68.7%).

Men and women had fairly similar compliance at each timepoint (Table 2). PRO-CTCAE compliance was slightly higher in the age group ≥ 60 years (week 12 cumulative, 78.5%) compared with the younger age group ≤ 59 years (week 12 cumulative, 68.9%). In contrast, EORTC compliance was relatively similar at each timepoint.

PRO-CTCAE compliance fluctuated on both systems, but cumulative compliance was similar (IVRS, 74.5%; Internet, 76.7%). In contrast, EORTC compliance was higher on IVRS (cumulative month 3 IVRS, 85.6%; Internet, 79.8%); however, the number of IVRS users was small.

### Patient and Staff Feedback

EOS questionnaires were completed by 192 (82.8%) of 232 patients (Internet, n = 157; IVRS, n = 35*)*. Among nonresponders (n = 40), 20 had not completed any electronic PRO-CTCAE questionnaires postbaseline (ie, passive withdrawers; IVRS, n = 3; Internet, n = 17) and 20 had engaged postbaseline (IVRS, n = 4; Internet, n = 16). Almost all patients found the system(s) easy to use (IVRS, n = 35 [100%]; Internet, n = 156 [98.1%]) and access (IVRS, n = 32 [94.1%]; Internet, n = 148 [93.1%]), felt prepared post-training (IVRS, n = 35 [100%]; Internet, n = 153 [96.8%]), and more than 80% of all patients (151 of 188) were happy completing weekly questionnaires. Qualitative comments echoed this usability. Time to complete was “about right” (IVRS, n = 29 [82.9%]; Internet, n = 157 [99.4%]), although 6 IVRS users felt it was slow or took “too long”. Approximately one third (62 of 184; 33.7%) reported missing a week mainly because of illness (n = 21) or forgetting (n = 24). Fourteen hormone therapy patients (14 of 29; 48.3%) found few of the symptom questions to be relevant to them.

Staff surveys (32 of 50; 64%) indicated positive views about regular/weekly symptom reporting. Despite some concerns over resources and the time needed to review data, the benefit for auditing symptoms and directing consultations and in clinical trials was emphasized.

### System Testing

During the 17-month study, 3,170 questionnaires were completed (2,413 PRO-CTCAE questionnaires and 757 EORTC questionnaires) and proportionately few issues were recorded—38 patient-related and 69 system-related issues. Notable system issues included reminder problems (n = 20), information technology/server issues (n = 7) affecting access or auto-emails, completion report issues (n = 12), and some technical difficulties (Internet, n = 11; IVRS, n = 8). Overall downtime was not systematically recorded, but isolated incidences were experienced. Patient issues included initial access problems (n = 19), completion queries (n = 8), and lost/forgotten username and/or password (n = 6). The PRO database was combined successfully with the standard clinical trial database with only a single issue during the data retrieval/combining process.

## DISCUSSION

To our knowledge, this is the first study illustrating the feasibility and acceptability of an electronic system to collect both self-reported PRO-CTAE and HRQOL data from a diverse sample of United Kingdom patients with cancer. The results demonstrate patient willingness, low attrition (5.2%; 13 of 249 active withdrawals), and high compliance (approximately 60% to 70% week 12; cumulative, approximately 75% to 80%). Most participants preferred the Internet, but approximately 17% of those who were older used IVRS, indicating an that alternative mode was desirable. Combining PRO data with the clinical trial database worked effectively with few system issues recorded. This approach is therefore recommended and a comparative evaluation is now required.

The 47.9% recruitment rate is lower than the 70% uptake rate routinely observed in PRO studies,^11^ but is encouraging compared with the 31% to 64% uptake rate in mailed cancer surveys.^26^ Target participants included various diagnoses, which made robust interpretation of any differences between cancer groups challenging as a result of small frequencies. Disease site was not associated with the rate of consent for the study; however, uptake was specifically low in some groups (eg, prostate and hormone therapy). Therefore, the study findings cannot be generalized to all cancer populations and may not reflect the compliance/views of specific cancer groups who were under-represented in this study. Patients required Internet access or a touch-tone telephone, which excluded some patients or those not confident with technology. In a clinical trial setting, offering paper-based PROs should continue, although this may diminish as Internet use increases. Those who declined tended to be older,^19^ but reasons focused on being too busy/overwhelmed with treatment. This study provided limited personal benefit beyond altruism; therefore, if electronic PRO reporting was integrated into treatment trials, uptake could be higher. Basch et al^18,27^ report higher uptake, although their approach included clinic-based computer completions. Future trials, combining remote/home-based completions with clinic-based completions may optimize compliance.

Compliance overall was high (approximately 60% to 70% at week 12; cumulative, approximately, 75% to 80%) and higher still in chemotherapy patients regularly going to the hospital for treatment reviews. PRO-CTCAE items chosen were particularly applicable to chemotherapy-related symptoms. Lower weekly compliance for surgical patients may be a result of the intense recovery period immediately postsurgery and less frequent review postsurgery. Some clinical trials that included paper-based QOL measures have observed higher compliance (75% at 6 months^28^), but the current study had more intense frequency (weekly/every 4 weeks). In an active clinical trial, compliance may be higher because of patient selection, trial-related incentives, and item relevance.^29^ Basch et al^27^ reported 92% cumulative compliance for home PRO-CTCAE reporting in trial patients, but 14.7% of questionnaires were collected via a coordinator directly ringing patients.

Despite most participants choosing to use the Internet, some older patients preferred the IVRS.^27^ An alternative real-time method is desirable if the purpose of collection is tracking symptoms/AEs, whereas if the aim is obtaining HRQOL outcomes, paper-based alternatives may suffice. Overall compliance did not differ across systems, but different patterns were observed, potentially influenced by the order of presentation: EORTC was presented first on the IVRS at the once every 4 weeks intervals (explaining higher EORTC IVRS compliance), whereas Internet users had to proactively choose both questionnaires. Furthermore, completions on the Internet were quicker, which may be a result of the systems themselves (ie, IVRS required the questions/answers to be read out at a comprehensible speed), but it may also reflect the younger, more educated Internet users.

Patient feedback was positive, finding the systems acceptable and problem free. Most issues were related to initial queries or access problems and were quickly resolved. Reasons for noncompletion were a result of being unwell or forgetting, rather than negativity about the systems.

The systems were only available in English, which is a limitation that should be addressed in future work. Furthermore, the general oncology setting influenced how the study was presented (focusing on system testing rather than on the value of collecting PROs in trials) and potentially affected uptake and compliance. The definition of baseline differed to what would be done in a formal clinical trial setup: A lenient baseline PRO-CTCAE/HRQOL completion (up to 3 days postregistration) was used, rather than prerandomization, as in many clinical trials.

The study highlights implementation considerations for future actual clinical trial use, including the need for a serious AE (or suspected unexpected serious adverse reaction) alert system, clinicians directly receiving symptom summary reports or alerts, and a function for individual symptom reporting or adding other free-text symptoms. Some of the considerations also relate to concerns voiced regarding the ethics of PRO data in clinical trials and the timescale of data review.^30^ Using an electronic PRO system with automated alerts helps with the timely management of these issues compared with paper-based methods, but creates new training needs for trial staff.^31^ Furthermore, electronic data collection requires continuous assessment to ensure that information technology systems and infrastructure meet clinical trial regulation and Medicines and Healthcare products Regulatory Agency and European Medicines Agency requirements.^32^

In conclusion, routine collection of PRO data in cancer clinical trials is increasingly important. This study illustrates the feasibility and patient acceptance of electronic PROs during treatment and incorporating PRO data into clinical trials. Additional exploration of this system is warranted where PROs are combined with other trial data—alongside traditional approaches—to improve data quality and patient safety.

## References

[1] Kluetz PG Kanapuru B Lemery S, et al: Informing the tolerability of cancer treatments using patient-reported outcome measures: Summary of an FDA and Critical Path Institute workshop. Value Health 21:742-747, 2018 29909880 10.1016/j.jval.2017.09.009

[2] Basch E Jia X Heller G, et al: Adverse symptom event reporting by patients vs clinicians: Relationships with clinical outcomes. J Natl Cancer Inst 101:1624-1632, 2009 19920223 10.1093/jnci/djp386 PMC2786917

[3] National Cancer Institute: Common Terminology Criteria for Adverse Events (CTCAE) v4.0. https://ctep.cancer.gov/protocolDevelopment/electronic_applications/docs/CTCAE_4.03.xlsx

[4] Basch E: The missing voice of patients in drug-safety reporting. N Engl J Med 362:865-869, 2010 20220181 10.1056/NEJMp0911494 PMC3031980

[5] Basch E Iasonos A McDonough T, et al: Patient versus clinician symptom reporting using the National Cancer Institute Common Terminology Criteria for Adverse Events: Results of a questionnaire-based study. Lancet Oncol 7:903-909, 2006 17081915 10.1016/S1470-2045(06)70910-X

[6] Pakhomov SV Jacobsen SJ Chute CG, et al: Agreement between patient-reported symptoms and their documentation in the medical record. Am J Manag Care 14:530-539, 2008 18690769 PMC2581509

[7] Di Maio M Gallo C Leighl NB, et al: Symptomatic toxicities experienced during anticancer treatment: Agreement between patient and physician reporting in three randomized trials. J Clin Oncol 33:910-915, 2015 25624439 10.1200/JCO.2014.57.9334

[8] Reeve BB Mitchell SA Dueck AC, et al: Recommended patient-reported core set of symptoms to measure in adult cancer treatment trials. J Natl Cancer Inst 106:dju129, 2014 25006191 10.1093/jnci/dju129 PMC4110472

[9] Di Maio M Basch E Bryce J, et al: Patient-reported outcomes in the evaluation of toxicity of anticancer treatments. Nat Rev Clin Oncol 13:319-325, 2016 26787278 10.1038/nrclinonc.2015.222

[10] Kluetz PG O’Connor DJ Soltys K: Incorporating the patient experience into regulatory decision making in the USA, Europe, and Canada. Lancet Oncol 19:e267-e274, 2018 29726391 10.1016/S1470-2045(18)30097-4

[11] Velikova G Booth L Smith AB, et al: Measuring quality of life in routine oncology practice improves communication and patient well-being: A randomized controlled trial. J Clin Oncol 22:714-724, 2004 14966096 10.1200/JCO.2004.06.078

[12] Kotronoulas G Kearney N Maguire R, et al: What is the value of the routine use of patient-reported outcome measures toward improvement of patient outcomes, processes of care, and health service outcomes in cancer care? A systematic review of controlled trials. J Clin Oncol 32:1480-1501, 2014 24711559 10.1200/JCO.2013.53.5948

[13] Basch E Deal AM Dueck AC, et al: Overall Survival Results of a Trial Assessing Patient-Reported Outcomes for Symptom Monitoring During Routine Cancer Treatment. JAMA 318:197-198, 2017 28586821 10.1001/jama.2017.7156 PMC5817466

[14] Denis F Lethrosne C Pourel N, et al: Randomized trial comparing a Web-mediated follow-up with routine surveillance in lung cancer patients. J Natl Cancer Inst 109:djx029, 2017 10.1093/jnci/djx029 28423407

[15] Basch E Reeve BB Mitchell SA, et al: Development of the National Cancer Institute’s Patient-Reported Outcomes version of the Common Terminology Criteria for Adverse Events (PRO-CTCAE). J Natl Cancer Inst 106:dju244, 2014 25265940 10.1093/jnci/dju244 PMC4200059

[16] Gwaltney C Coons SJ O’Donohoe P, et al: “Bring your own device” (BYOD): The future of field-based patient-reported outcome data collection in clinical trials? Ther Innov Regul Sci 49:783-791, 2015 30222388 10.1177/2168479015609104

[17] Basch E Artz D Dulko D, et al: Patient online self-reporting of toxicity symptoms during chemotherapy. J Clin Oncol 23:3552-3561, 2005 15908666 10.1200/JCO.2005.04.275

[18] Basch E Dueck AC Rogak LJ, et al: Feasibility assessment of patient reporting of symptomatic adverse events in multicenter cancer clinical trials. JAMA Oncol 3:1043-1050, 2017 28208174 10.1001/jamaoncol.2016.6749 PMC5553624

[19] Office of National Statistics: Internet access–households and individuals, Great Britain: 2019. https://www.ons.gov.uk/peoplepopulationandcommunity/householdcharacteristics/homeinternetandsocialmediausage/bulletins/internetaccesshouseholdsandindividuals/2019#9-in-10-adults-use-the-internet-at-least-weekly

[20] Banerjee AK Okun S Edwards IR, et al: Patient-reported outcome measures in safety event reporting: PROSPER Consortium guidance. Drug Saf 36:1129-1149, 2013 24092596 10.1007/s40264-013-0113-z PMC3834161

[21] Thanarajasingam G Minasian LM Baron F, et al: Beyond maximum grade: Modernising the assessment and reporting of adverse events in haematological malignancies. Lancet Haematol 5:e563-e598, 2018 29907552 10.1016/S2352-3026(18)30051-6 PMC6261436

[22] Groenvold M Aaronson NK Darlington AE, et al: Focusing on core patient-reported outcomes in cancer clinical trials: Letter. Clin Cancer Res 22:5617, 2016 28151714 10.1158/1078-0432.CCR-16-1529

[23] Lancaster GA Dodd S Williamson PR: Design and analysis of pilot studies: Recommendations for good practice. J Eval Clin Pract 10:307-312, 2004 15189396 10.1111/j..2002.384.doc.x

[24] Aaronson NK Ahmedzai S Bergman B, et al: The European Organization for Research and Treatment of Cancer QLQ-C30: A quality-of-life instrument for use in international clinical trials in oncology. J Natl Cancer Inst 85:365-376, 1993 8433390 10.1093/jnci/85.5.365

[25] Holch P Warrington L Bamforth LCA, et al: Development of an integrated electronic platform for patient self-report and management of adverse events during cancer treatment. Ann Oncol 28:2305-2311, 2017 28911065 10.1093/annonc/mdx317 PMC5834137

[26] VanGeest JB Johnson TP: Using incentives in surveys of cancer patients: Do “best practices” apply? Cancer Causes Control 23:2047-2052, 2012 23076587 10.1007/s10552-012-0082-z

[27] Basch E Dueck AC Rogak LJ, et al: Feasibility of implementing the Patient-Reported Outcomes version of the Common Terminology Criteria for Adverse Events in a multicenter trial: NCCTG N1048. J Clin Oncol 36:3120-3125, 2018 10.1200/JCO.2018.78.8620 PMC6209091 30204536

[28] Jayne D Pigazzi A Marshall H, et al: Effect of robotic-assisted vs conventional laparoscopic surgery on risk of conversion to open laparotomy among patients undergoing resection for rectal cancer: The ROLARR randomized clinical trial. JAMA 318:1569-1580, 2017 29067426 10.1001/jama.2017.7219 PMC5818805

[29] Sandler KA Mitchell SA Basch E, et al: Content validity of anatomic site-specific Patient-Reported Outcomes version of the Common Terminology Criteria for Adverse Events (PRO-CTCAE) item sets for assessment of acute symptomatic toxicities in radiation oncology. Int J Radiat Oncol Biol Phys 102:44-52, 2018 30102201 10.1016/j.ijrobp.2018.04.048

[30] Kyte D Ives J Draper H, et al: Management of patient-reported outcome (PRO) alerts in clinical trials: A cross sectional survey. PLoS One 11:e0144658, 2016 26785084 10.1371/journal.pone.0144658 PMC4718453

[31] Mercieca-Bebber R Calvert M Kyte D, et al: The administration of patient-reported outcome questionnaires in cancer trials: Interviews with trial coordinators regarding their roles, experiences, challenges and training. Contemp Clin Trials Commun 9:23-32, 2017 29696221 10.1016/j.conctc.2017.11.009 PMC5898562

[32] Medicines & Healthcare Products Regulatory Agency: Guidance: Medical device stand-alone software including apps (including IVDMDs). https://www.gov.uk/government/uploads/system/uploads/attachment_data/file/564745/Software_flow_chart_Ed_1-02.pdf

